# Closed-Loop Pharmacologic Control of Blood Pressure: A Review of Existing Systems

**DOI:** 10.7759/cureus.45188

**Published:** 2023-09-13

**Authors:** Temur Baykuziyev, Muhammad Jaffar Khan, Arunabha Karmakar, Muhammad Arif Baloch

**Affiliations:** 1 Anesthesiology and Critical Care, Hamad Medical Corporation, Doha, QAT

**Keywords:** artificial intelligence and robotics in healthcare, intensive & critical care, perioperative period, vasopressors, blood pressure management, closed-loop system

## Abstract

Blood pressure management is a critical aspect of patient care, particularly in surgical and critical care settings. Closed-loop systems, which utilize real-time data and feedback to adjust treatment interventions, have gained attention for their potential to enhance blood pressure control. This review explores the application of closed-loop systems in blood pressure management. We discuss various closed-loop approaches, including their mechanisms, benefits, and limitations. By harnessing real-time patient data and feedback, closed-loop systems can tailor interventions dynamically, thus enhancing blood pressure regulation. Additionally, we examine the integration of advanced monitoring technologies and artificial intelligence algorithms in closed-loop systems. The review highlights recent studies and their findings, emphasizing the evolving landscape of closed-loop blood pressure management across different clinical scenarios. From the perioperative period to critical care settings, closed-loop systems hold the potential to optimize patient outcomes by precisely adjusting vasopressor administration in response to continuous blood pressure fluctuations. By providing insights into the current state of closed-loop systems for blood pressure control, this review offers a comprehensive overview of their potential contributions to improved patient outcomes and future directions for research and implementation.

## Introduction and background

With the advent of autonomous systems, a lot of research has been dedicated to the development of closed-loop self-driving systems for drugs to maintain blood pressure (BP).

Normal human physiology tends to self-regulate blood pressure by reflexes mediated by hemodynamic centers in the brain stem. This mechanism is disrupted in pathological states such as traumatic brain injury, critical illness, or states of controlled physiology such as under general anesthesia.

**Figure 1:**
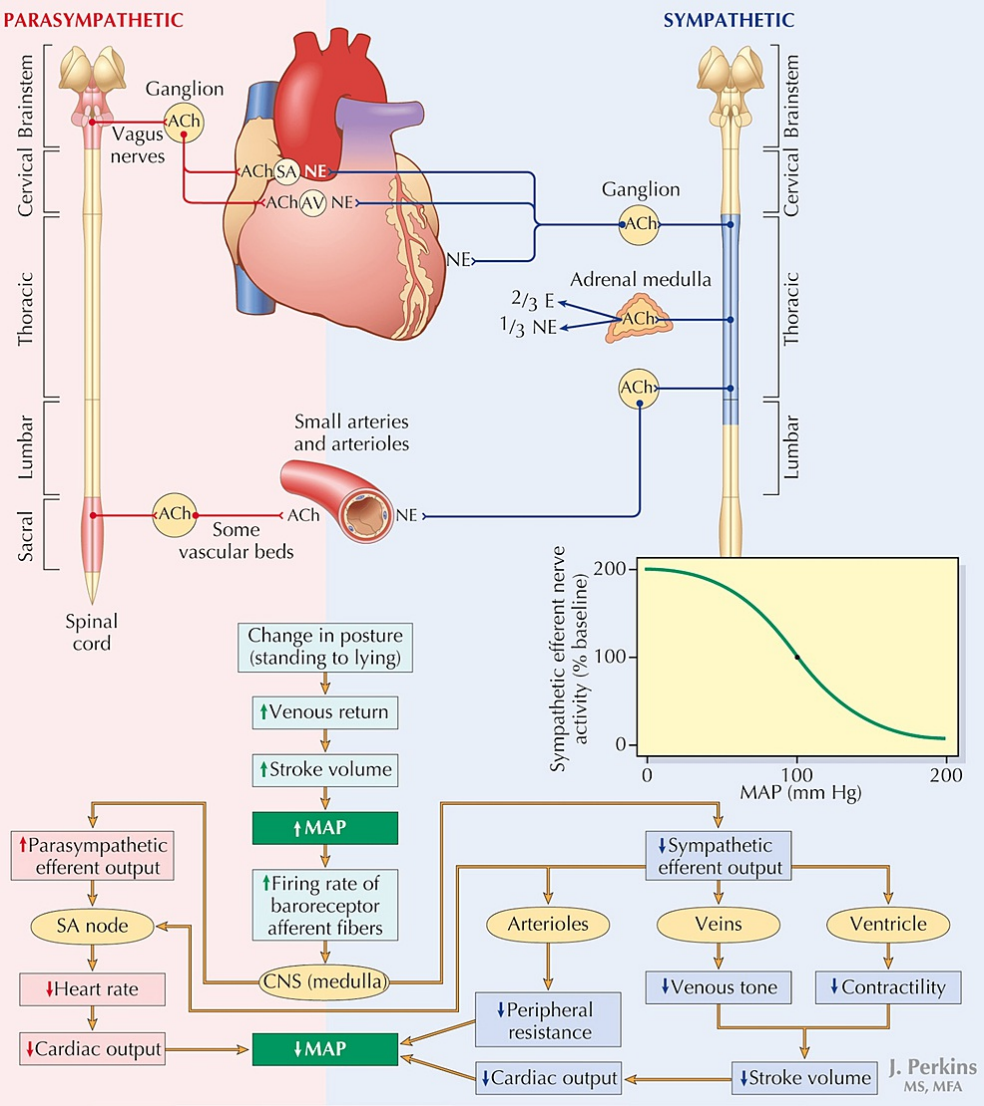
Autonomic Nervous System and Arterial Baroreceptor Reflexes. Both sympathetic and parasympathetic nerves innervate the sinoatrial (SA) and atrioventricular (AV) nodes. The myocardium is innervated by sympathetic nerves. Arterial and venous vessels throughout most of the body are innervated by sympathetic nerves, whereas the parasympathetic nervous system innervates vessels in the genital organs and gastrointestinal tract. Autonomic efferent activity is regulated by the baroreceptor reflex, in response to changes in arterial pressure detected at the carotid sinus and aortic arch baroreceptors. The response to a change in posture is illustrated. ACh, acetylcholine; AV, atrioventricular; CNS, central nervous system; E, epinephrine; MAP, mean arterial pressure; NE, norepinephrine; SA, sinoatrial. Reproduced with permission from reference [1]. Copyright 2013, Elsevier

Organ and tissue perfusion depends on the flow of blood and the pressure gradient between the afferent (mean arterial pressure (MAP)) and efferent vessels (tissue venous pressure). For any given state, therefore, tissue perfusion is proportionate to MAP. Exceptions to this rule exist, for example, coronary artery perfusion which depends on aortic diastolic pressure. Reduced MAP leads to tissue ischemia and increased MAP can cause bleeding. To prevent these, rapid in vivo control of blood pressure is mediated by the autonomic nervous system (Figure 1), so named for its autonomic self-regulating nature. Unique biological feedback mechanisms act as drivers and inhibitors for this system and try to maintain physiologic parameters in an optimum range. These feedback mechanisms have been studied and are well elucidated in modern physiology. Similar ex vivo systems are possible considering the same feedback signals that drive the human autonomic nervous system.

Having a closed-loop autonomic system to maintain blood pressure allows for a far better beat-to-beat control of BP. These machines are better at observing and analyzing biodata and are robust at interventions as per their programming. Additionally, they are non-fatigable. Physicians looking after patients are also free to focus on other aspects of care.

Design of a biological system to maintain blood pressure

Mean arterial pressure (MAP) is a product of blood flow (cardiac output (CO)) and systemic vascular resistance (SVR).

MAP = CO * SVR

To maintain MAP in an optimum range, either CO or SVR must be altered. In general, CO is dependent on contractility and end-diastolic volume i.e., inotropy and pre-load. Pre-load depends on a person’s circulating volume. Systemic vascular resistance depends on vascular tone which is activated by endogenous catecholamines. In disease, the body’s ability to maintain these are affected. Physicians in the emergency department, perioperative care, and critical care departments play an important role in regulating acute changes in a patient’s blood pressure.

**Figure 2:**
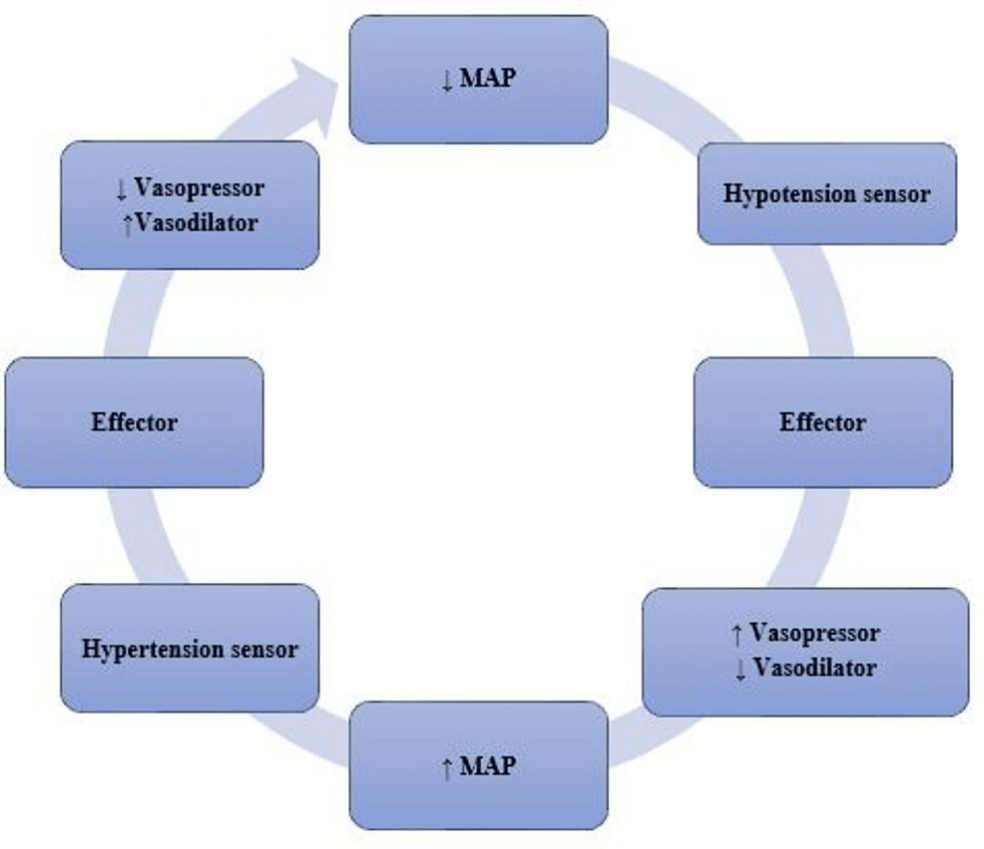
A Basic Controlled-Loop System to Maintain Blood Pressure. A single measured variable (MAP) acts as feedback followed by two system changes: a change in vasopressor and a change in vasodilator that together act to maintain MAP in a normal range. MAP, mean arterial pressure

Vasoactive pharmacologic agents act on the peripheral vessels to increase vascular tone (vasopressor) or reduce it (vasodilator). If CO remains the same, MAP is directly proportional to SVR. A basic control system to control SVR would thus be as shown in Figure 2.

**Figure 3:**
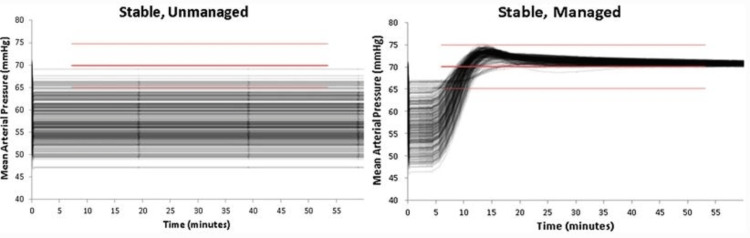
Closed-Loop System Reaching Equilibrium. Depending on the speed of detection and response, the system can reach an equilibrium state with MAP maintained at or very close to a normal range. MAP, mean arterial pressure Reproduced with permission from reference [2]. Copyright 2018, Springer.

In a closed loop, a controller observes specific system variables and provides corresponding interventions to regulate the system, following a predefined algorithm. Furthermore, the system (Figure 3) should reach an equilibrium state with MAP maintained at or near a normal range [2]. 

The necessity of closed-loop blood pressure management

The Clinical Setting

Blood pressure allows adequate driving force for organ perfusion. Normotension is necessary to maintain an adequate homeostatic environment for cellular processes to function. Low blood pressure reduces perfusion thereby disrupting the supply and clearance of vital substrates and oxygen to cells causing apoptosis. On a tissue level, ischemia results causing organ dysfunction, which if left unchecked can cause gangrene. Normal homeostatic processes maintain blood pressure in an awake, non-diseased individual. The same individual has limited capability to control their BP under disease state or under anesthesia including general and neuraxial. Timely BP management thus plays a vital role in the perioperative period to improve surgical outcomes as well as during critical illness in the ICU. Disturbed physiology causing impaired regulation of BP also occurs in accidents and trauma victims arriving in the emergency. Hypotension has been associated with organ dysfunction and adverse myocardial, cerebral, and renal outcomes [3,4].

The Provider

Emergency physicians, anesthesiologists, and critical care physicians look after patients who may develop hypotension or hypertension and associated organ dysfunction during their hospital stay. These providers in addition need to assess, administer, treat, and monitor a wide variety of parameters such as oxygen saturation, fluid balance, electrolyte disturbances, pain control, sedation, and interpersonal communication. Automation of tasks can enable physicians to prioritize other tasks and improve patient outcomes. Additionally, autonomous systems are better at observing, calculating, and dosing and are less prone to human error. Additionally, they are non-fatigable.

In this review, we will focus on the use of closed-loop blood pressure management systems in the perioperative period and critical illness period.

## Review

Basic requirements of closed-loop control of BP management

Effective beat-to-beat control of a patient’s BP requires 1) a monitor capable of beat-to-beat measurement and display of blood pressure (such as an arterial line); 2) A pathological state causing BP changes secondary to changes in vasomotor tone with minimal influences from circulating volume and inotropy; 3) Either boluses or continuous Infusion of rapidly titratable vasopressor or vasodilator medications (Fast onset and short duration).

Early studies using closed-loop systems for BP

R.V. Jackson and colleagues described in 1977 how a controlled infusion of sodium nitroprusside was used to treat otherwise refractory malignant hypertension [5]. They used a microprocessor into which a very simple algorithm to calculate the rate of infusion was programmed.

R = K1 + K2e

R: Rate of infusion

K1: Infusion rate corresponding to desired mean blood pressure when stable control has been achieved

K2: Constant of proportionality or gain. (A value of five was obtained experimentally.)

e: Error term which represents the difference between the desired blood pressure and the actual blood pressure (mmHg)

As per the terms described above, the desired target of the algorithm was that K2e should approach zero thus allowing R to equal K1. The microprocessor was an 8-bit device. Its instructions were altered by way of a visual display unit (VDU) or teletype. The teletype allowed the generation or loading of paper tape programs. Programming was in machine code. The digitized mean BP was loaded into a memory register every two seconds via a microprocessor input/output port. Comparison of actual BP with desired BP allowed “e” to be calculated.

Initially, an approximate value of K1 was entered into the control algorithm. K1 was incremented or decremented after each BP sample, depending on whether the BP exceeded or not attained the desired value.

In this case, the blood pressure was maintained around the target range. Brief interruptions of sodium nitroprusside (SNP) infusion were done to evaluate if a patient would respond to oral anti-hypertensive therapy. Thiocyanate concentrations were measured as an index of toxicity. After six days, blood pressure control was maintained with oral therapy alone.

**Figure 4:**
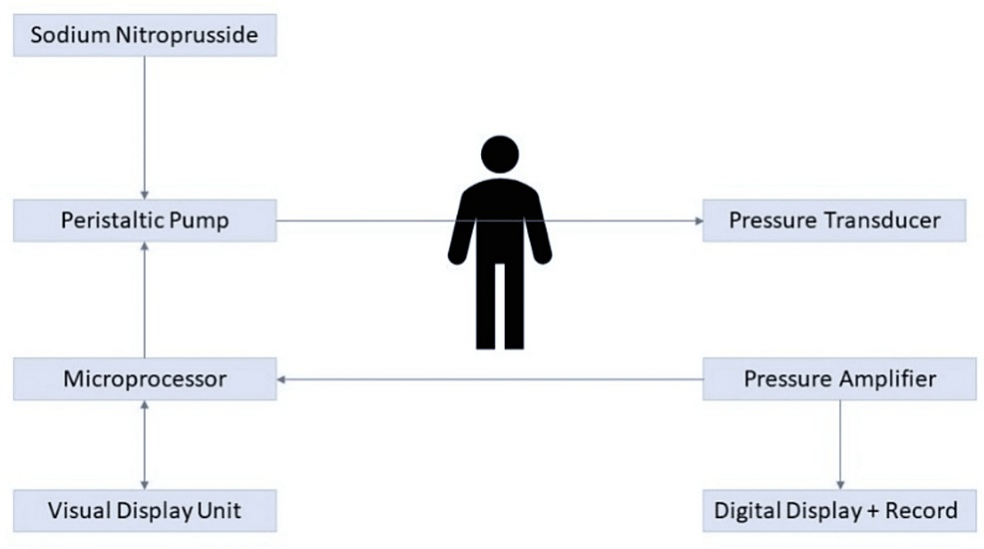
Closed-Loop System. The system used by R.V. Jackson and colleagues for controlling BP with sodium nitroprusside and a microprocessor. Reproduced with permission from reference [5]. Copyright 1977, Wiley.

The above-described successful management of malignant hypertension with SNP and successful transition to oral therapy was performed with a microprocessor system whose minimal requirements were a pressure transducer and amplifier, a microprocessor, a teletype, and a visual display unit (Figure 4).

With the improvement of microprocessor technology, more robust systems were developed in the coming years focused on BP control.

Yasuhiro Fukui and colleagues conducted two experiments - one using a hybrid computer model and the second using six dogs, using increasing or decreasing halothane concentration to control MAP using a discrete digital control system [6]. The delivered concentration of halothane was controlled automatically to maintain MAP at a desired level by using a discrete time-optimal control algorithm. They also showed the feasibility of using a hybrid computer model of the uptake and distribution of halothane in the testing of the control algorithm thus eliminating the need to use animals and anesthesia machines in the preliminary experiments. The use of a discrete digital control system was an improvement over the conventional analog approach. It was easier to implement multivariable controls, sequential controls (start-up, shut down, or emergency operation), adaptive control, and overall supervisory control. The study authors recognized that a single variable could not be used to control halothane concentration as there would be multiple causes for changing BP which might require action other than altering halothane concentration. It was also able to use both MAP and halothane concentrations in deciding what concentration to use the next time. However, the authors also advised a few system checks. Problems may arise, for example, if a clot blocking the arterial cannula gives a falsely low reading. The anesthesia machine could inadvertently reduce the halothane concentration to low levels. Additionally, if there was an artefactually high reading, it would deliver a very high concentration. Thus, alarms and fail checks need to be made a part of the system.

In the modern age, systems have advanced to the point of incorporation of artificial intelligence (AI) and machine learning to be far more robust and capable of analyzing multiple patient parameters so as to even predict hypotension before it occurs [7,8].

Use of closed-loop vasopressor systems in the perioperative period

Intraoperative hypotension is commonly observed during surgical procedures and is likely to exert a negative influence on postoperative outcomes [9-11]. Several researchers have described a significant association between episodes of low blood pressure and unfavorable events following surgery, encompassing complications like myocardial injury, acute kidney dysfunction, strokes, and even mortality [3,4,12-16]. The extent and duration of hypotension seem to influence the occurrence of these complications. Interestingly, even just one minute with a MAP below 55 to 65 mmHg during surgery has been associated with a heightened risk of adverse outcomes [11]. 

Notably, a multicentre randomized controlled trial by Futier et al. demonstrated a promising approach to mitigate these risks [17]. Their study showed that maintaining an "individualized" arterial pressure during surgery, within 10% of the patient's baseline reference value, led to a reduction in postoperative organ dysfunction compared to standard management. This was accomplished through the continuous administration of norepinephrine, initiated at the induction of anesthesia and maintained until the conclusion of the surgical intervention. It is important to note, however, that the use of vasoconstrictors to maintain tailored arterial pressure might obscure the development of hypovolemia, potentially putting patients at risk due to compromised end-organ perfusion. 

Achieving an ideal balance of flow and pressure involves ongoing assessment of these factors and adhering to established protocols for administering vasopressors and fluids. This approach, known as "individualized hemodynamic management," has demonstrated a clear advantage over standard care by reducing postoperative complications [18]. 

Additionally, hypotension emerges as a delayed clinical signal of underlying hemodynamic instability after exhaustion of compensatory mechanisms. To tackle these challenges, the integration of artificial intelligence has emerged as a solution to facilitate the early identification of hypotension and, in certain scenarios, even predict its occurrence to support prompt interventions. Machine learning, a discipline of computer science dedicated to analyzing extensive datasets and developing predictive models, exhibits clear relevance within the realm of healthcare. Employing artificial intelligence to forecast hypotension based on hemodynamic data represents an innovative and captivating strategy. 

The Acumen^TM^ Hypotension Prediction Index (HPI) software, developed by Edwards Lifesciences in Irvine, CA, USA, employs artificial intelligence, specifically machine learning techniques, to predict hypotension based on distinctive features found within blood pressure waveforms [7,8]. The HPI is a numerical value, devoid of units, ranging from 0 to 100. It quantifies the likelihood of a patient encountering hypotension, characterized by a mean arterial pressure (MAP) dipping below 65 mmHg for a minimum of one minute. Elevated HPI values correspond to an elevated probability of imminent hypotension. Notably, an HPI exceeding 85 triggers auditory and visual alerts, accompanied by a pop-up notification and a supplementary screen. This secondary screen presents advanced hemodynamic parameters, empowering clinicians to delve into the reasons behind the impending hypotensive event and initiate relevant therapeutic interventions. Nevertheless, effectively and precisely maintaining optimal mean arterial pressure (MAP) and cardiac output is no small feat during complex surgeries. This demands frequent manual adjustment to vasopressor infusion rates and timely fluid delivery, requiring the unwavering focus of healthcare providers. Yet, maintaining this continuous attention can pose a significant challenge in the context of the demanding surgical environment [19]. Computer systems that can automatically adjust vasopressor medications to reach specific target blood pressure levels could potentially enhance the management of patients' blood pressure [2,20,21]. In critical care settings like intensive care units, surgical units, and emergency rooms, where high-stress situations can create challenges for clinicians, closed-loop vasopressor systems can handle labor-intensive tasks. They improve the timing, reliability, and consistency of therapy, reducing the chance of human errors. Consequently, closed-loop systems offer an advantage in enhancing the existing standard of care in critical care settings.

**Figure 5:**
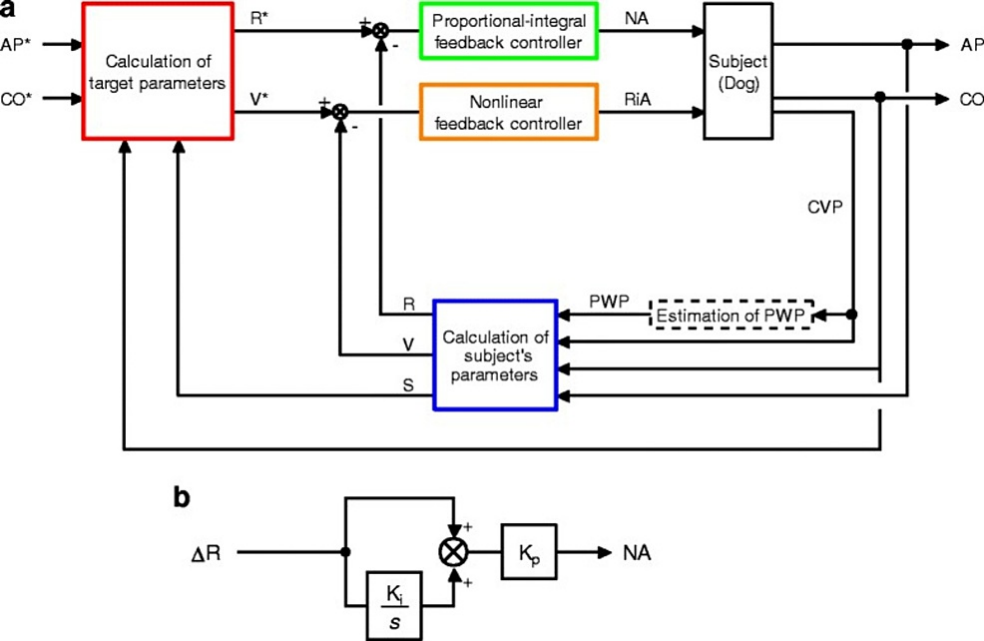
Closed-Loop Drug Infusion System for Automated Hemodynamic Resuscitation in Septic Shock. a). Block diagram of the system for simultaneous control of mean arterial pressure (AP) and cardiac output (CO) in septic shock. AP* and CO* represent target AP and target CO, respectively. From these target variables, target values of systemic arterial resistance (R*) and stressed blood volume (V*) are determined (red rectangle). Subject’s R, V, and Frank-Starling slope of the left ventricle (S) are calculated from AP, CO, central venous pressure (CVP) and pulmonary wedge pressure (PWP) (blue rectangle). The proportional-integral controller adjusts infusion rate of noradrenaline (NA) to minimize the difference between R and R* (green rectangle). A non-linear controller adjusts infusion of Ringer’s acetate (RiA) to minimize the difference between V and V* (orange rectangle). b). Block diagram of the proportional-integral controller. Ki and Kp represent the integral and proportional gain constants, respectively. s is a Laplace operator. Reproduced with permission from reference [23]. Copyright 2017, Springer Nature.

A study, conducted by Ribeiro Marques and colleagues, described a new approach to managing blood pressure [22]. They used a special automated system that used a vasopressor called phenylephrine to treat normovolemic vasodilation induced by sodium nitroprusside. They tested this system on pigs and compared the performance of this system to physicians managing the same condition in a research study. They hypothesized that the automated system would keep the blood pressure at a certain level more accurately and with less use of the medication. Their results showed that the system, controlled blood pressure more precisely than the doctors did. However, because of the small number of cases, the study wasn't strong enough to prove this difference statistically. Still, the system was effective in preventing low blood pressure, just like physicians, but additionally, it caused fewer instances where blood pressure went too high. It's important to note that the doctors in the study only had one task, which was managing blood pressure, without any of the other distractions and responsibilities that come with regular anesthesia care. Similarly, a computer-controlled closed-loop system (Figure 5) for automated hemodynamic resuscitation in septic shock was developed and evaluated in a canine model by Uemura et al. [23]. The system effectively maintained target arterial pressure and cardiac output using noradrenaline and Ringer's acetate solution, even with less invasive measurements. The approach showed promise for precise patient care in septic shock. However, there are certain limitations to this study. The authors used a previously developed method to estimate Pulmonary Wedge Pressure (PWP), but its accuracy hasn't been confirmed in endotoxemia subjects. Endotoxin's impact on pulmonary artery and vein mechanics might affect the reliability of this estimation method. Further research is needed in these areas. Endotoxin is often used in animal models of sepsis, though it doesn't entirely simulate human sepsis. Differences in time course and bacterial type are concerns. Ringer’s acetate infusion early in system activation reduced arterial resistance, which could potentially lead to system malfunction. Severe myocardial depression in septic shock patients could impact the system's effectiveness, necessitating additional control loops. Applying for hemodynamic support an hour after symptom onset may not reflect real-world early intervention. Another limitation is the absence of splenectomy or plasma volume assessment tools, which could have clarified the rise in haematocrit. 

Closed-loop vasopressor system and its role in obstetrics

Two earlier trials provided data comparing the use of automated closed-loop systems for administering vasopressors (phenylephrine or ephedrine) with manual control in patients scheduled for elective cesarean sections under spinal anesthesia [24,25]. In the first study, the authors employed an automated system to manage phenylephrine administration, focusing on controlling arterial pressure (AP). They calculated the proportion of systolic blood pressure that was within ±20% of the baseline value. Compared to manual control, the utilization of automated systems resulted in an increased number of measurements falling within the desired range (odds ratio, 1.44; 95% CI, 1.04-2.0; I2 = 0.77, P < 0.0001). Additionally, there was no significant difference in the occurrence of episodes of hypotension or hypertension between the manual and automated control groups. In the second trial, the researchers employed an automated closed-loop system involving two vasopressors. Phenylephrine was used to address hypotension, while ephedrine was used for low arterial pressure (AP) with bradycardia. They focused on non-invasive beat-to-beat systolic AP as the controlled parameter. In this study, the group that was managed manually exhibited a notably greater number of patients experiencing hypotension (P = 0.001). 

Ngan Kee et al. compared the performance of two different closed-loop techniques (boluses versus continuous infusion) for the administration of phenylephrine [26]. 214 healthy patients undergoing cesarean deliveries with spinal anesthesia were included in the study. They found that the use of intermittent boluses resulted in more precise control of blood pressure and less total drug administration as compared with continuous infusion when using closed-loop control guided by checking blood pressure every minute with a cuff. The importance of this study lies in two main points. Firstly, the automated system for giving the medication worked well with this non-invasive blood pressure monitoring, and secondly, giving the medication in small, computer-guided boluses may work better than giving it continuously. Moreover, this study included one of the largest groups of patients where computer-assisted blood pressure management had been tested. Additionally, the same team compared the performance of norepinephrine versus phenylephrine in this system [27]. They analyzed data from a trial involving 104 elective cesarean section patients under spinal anesthesia. The computer-controlled system aimed to sustain baseline-like systolic blood pressure until fetal delivery. The result showed that norepinephrine demonstrated smaller errors in maintaining blood pressure, indicating better precision than phenylephrine. 

Hence, data from these trials suggest that arterial blood pressure is controlled better with a closed-loop system. Typically, these systems utilize non-invasive blood pressure measurements to calculate phenylephrine infusion rates during the perioperative period, but they are limited by the influence of movement and shivering artifacts on blood pressure readings. In the future, research should explore innovative methods to predict patients susceptible to spinal hypotension, enabling the customization of vasopressor treatments based on individual risk profiles.

Closed-loop vasopressor system in moderate to high-risk surgeries

**Figure 6:**
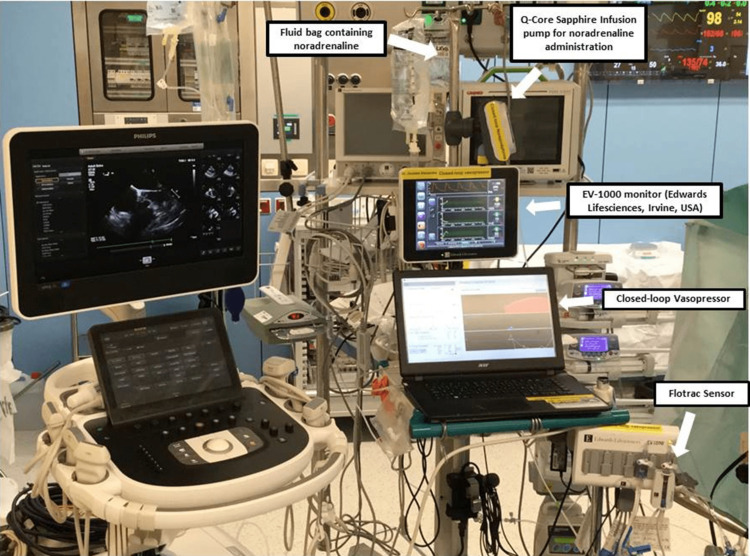
Closed-Loop Vasopressor System. The system with its different components used by Joosten and his colleagues in Erasme Hospital, Brussels, Belgium, during a cardiac case. Reproduced with permission from reference [28]. Copyright 2019, Elsevier.

In a first-in-man study, Joosten et al. developed a closed-loop vasopressor (CLV) controller (Figure 6) to investigate its feasibility in patients undergoing moderate and high-risk surgery [28]. They conducted a preliminary study involving 20 surgical patients. The CLV system, using norepinephrine infusion adjusted automatically based on continuously monitored arterial pressure, effectively maintained blood pressure within the target range for most of the surgery. Hypotension occurred for a very limited time, and there were no significant complications. This study indicates the potential of CLV control in minimizing hypotension during surgery. However, there were certain limitations of this study. Firstly, the CLV system was tested on a small number of patients, and it was a single-center study. Secondly, this study had a pump communication error that needed to be resolved. Thirdly, the behavior of this CLV system was not tested in conditions marked by more acute changes in hemodynamics. Finally, given the simultaneous requirement of fluids and vasopressors in high-risk surgical and ICU patients, this study could not evaluate the intricate interactions between these two treatment modalities.

Joosten et al. also investigated the role of the CLV system, guided by continuous non-invasive blood pressure, in high-risk patients undergoing renal transplant surgery [29]. In this study, the use of non-invasive CLV technology assisted clinicians in better adhering to blood pressure targets for patients without invasive arterial catheters. However, before this technology can be widely used in clinical settings, certain safety aspects needed to be addressed. These considerations include monitoring errors (like accidental disconnection or miscalibration), potential component errors (such as pump malfunctions), and the possibility of human error (setup of the system with the wrong drug). 

Furthermore, Joosten and his colleagues conducted a randomized controlled trial comparing automated closed-loop to manually controlled norepinephrine infusion [30]. This study aimed to assess whether the CLV controller was superior to traditional manual vasopressor management in minimizing hypotension during abdominal surgery. They included thirty patients undergoing elective intermediate-to-high-risk abdominal surgery, randomly dividing them into two groups. The CLV group employed the automated CLV controller, adjusting norepinephrine infusion based on MAP values obtained from an advanced hemodynamic device. In the control group, hypotension management involved manual norepinephrine adjustment. The results showed that the CLV group experienced significantly less time with hypotension during surgery compared to the control group (1.6% vs. 15.4%, respectively). Moreover, the CLV group spent notably less time with MAP below 65 mm Hg (0.2% vs. 4.5%). In conclusion, among patients undergoing intermediate- to high-risk surgeries under general anesthesia, the computer-assisted norepinephrine infusion adjustment through the CLV system reduced the incidence of hypotension compared to manual control. This study encountered certain limitations such as a small sample size, single-center study, and included patients were intermediate-to high-risk abdominal surgery. The results of this study cannot be extrapolated to other types of surgery or critical care settings. Thus, a large randomized-control trial is needed to evaluate its impact on patient outcomes. 

Individualized hemodynamic management, formerly known as "goal-directed hemodynamic therapy," has displayed enhanced patient outcomes compared to standard care during major surgeries, as recently validated by two meta-analyses [31,32]. , Joosten et al. employed a closed-loop system in a prospective randomized trial involving 38 intermediate- to high-risk patients undergoing abdominal or orthopedic surgery [33]. This system adjusted a norepinephrine infusion based on invasive arterial pressure monitoring, while a separate decision support system utilized mini-fluid challenges. This approach significantly reduced the duration of intraoperative time where mean arterial pressure fell below 90% of patients' preoperative baseline values, in comparison to the manual application of the same technique. In essence, the study showcased the effectiveness of this automated approach in maintaining blood pressure within a desired range throughout the surgical procedure, ensuring better hemodynamic stability for patients.

Rinehart et al. introduced a CLV controller to improve hypotension correction in postcardiac surgery patients [34]. They hypothesized patients managed with this system would experience less hypotension than those under standard care. In a 2-hour study involving 40 ICU patients after cardiac surgery, CLV-controlled norepinephrine infusion maintained mean arterial pressure between 65 and 75 mmHg. The CLV group had significantly lower hypotension (1.4% vs. 12.5%), more time within the target range (95% vs. 66%), and fewer instances of excessive pressure (3.2% vs. 20.6%) compared to controls. CLV led to more infusion rate adjustments but no adverse events. CLV effectively curbed postoperative hypotension. 

While closed-loop vasopressor therapy has shown its effectiveness when targeting baseline mean arterial pressure (MAP), limited information exists regarding its performance when systolic arterial pressure (SAP) is the controlled variable. Therefore, Rinehart et al. conducted a prospective cohort feasibility study with 12 patients undergoing intermediate- to high-risk abdominal surgery [35]. A computer-controlled closed-loop system administered norepinephrine infusion, targeting SAP at 130 mmHg. The closed-loop system delivered norepinephrine infusion for a median of 94.6% (25th-75th percentile: 90.0-98.0%) of the case time. The closed-loop vasopressor system effectively reduced intraoperative hypotension and maintained SAP within a 10% range of the target for over 90% of the case time. 

Closed-loop vasoactive system in the critical care unit

As previously mentioned, vasopressor therapy plays a pivotal role in maintaining hemodynamic stability in critically ill patients. Vasopressors, such as norepinephrine and vasopressin, are frequently administered to maintain adequate perfusion and prevent organ failure. However, the manual titration of vasopressors is often limited by variations in clinical assessments and time-consuming adjustments, potentially leading to under- or overtreatment. Ideally, adjustments in vasopressor infusion rates should promptly correspond to fluctuations in blood pressure readings. Nevertheless, accomplishing such a finely tuned feedback loop poses challenges.

Rinehart J. and his colleagues analyzed MAP data from 3623 patients (2530 from the ORs and 1093 from the ICU) who were on vasopressors. The coefficient of variations {= (standard deviation/mean value) *100} of MAP were 13.7 ± 5.4% and 18.4 ± 9.8% in the OR and ICU, respectively [19]. Patients on vasopressors spent 48.8% of treatment time with a MAP between 60 and 80 mmHg (11.2% time with MAP < 60 mmHg, and 40% with MAP > 80 mmHg). These findings offer a solid foundation for assessing whether a closed-loop vasopressor administration system can potentially achieve a state of "reduced variability." Closed-loop systems, integrating real-time patient data and advanced control algorithms, offer a solution to optimize vasopressor therapy and improve critical care patient outcomes [36]. 

High-fidelity mathematical models are being developed to replicate the cardiovascular (CV) responses of a critically ill patient with vasoplegic shock-induced hypotension to vasopressor therapy. They can be incorporated into closed-loop control algorithms for autonomous vasopressor administration once it is extensively validated [37]. 

Several studies have investigated the impact of closed-loop vasopressor administration on patient outcomes. Evidence suggests that closed-loop systems can lead to more stable hemodynamics, reduced variability in blood pressure, and decreased incidence of hypotensive and hypertensive episodes compared to manual titration. These systems have also been associated with shorter durations of vasopressor use and reduced healthcare resource utilization [38]. 

In a prospective cohort study conducted by Rinehart J. et al., a comparison was made between the performance of a group of anesthesia providers and a closed-loop management system known as the Learning Intravenous Resuscitator (LIR). This study utilized a simulated hemorrhage scenario with cardiac output monitoring. The results demonstrated that the closed-loop system maintained more consistent hemodynamics compared to the practitioners. This was attributed to the closed-loop system administering fluids earlier in the protocol and optimizing cardiac output before the onset of hemorrhage. In contrast, the practitioners tended to initiate resuscitation only after substantial hemodynamic shifts indicated the necessity for intervention [39]. 

A single-center, two-arm, parallel-group, randomized controlled superiority study concluded that closed-loop control of norepinephrine infusion significantly decreases postoperative hypotension compared to manual control in patients admitted to the ICU after cardiac surgery [34]. 

Utilizing a fuzzy logic algorithm for closed-loop control, which restricts the amount of norepinephrine exposure, could potentially maintain the efficacy and reservoir of α1-receptors. As a result, this approach might contribute to reducing patient resistance toward norepinephrine infusion. Thus, a closed-loop system based on fuzzy logic algorithm results in the use of much lower norepinephrine doses during weaning in patients with septic shock and leads to a decrease in the duration of weaning duration [40]. The Monte Carlo simulation study has shown that the closed-loop vasopressor controller remained effective in simulated patients exhibiting 0.1-10 × the expected population drug response [41]. 

Multiple internal fuzzy controllers can be structured in one system, each responsible for calculating the appropriate dosage of a medication. A system proposed by N. Sprunk et al. underwent testing within a simulation environment that mimics a canine cardiovascular system and its reactions to various medications [42]. The vasoactive drugs employed include isosorbide dinitrate, norepinephrine, dopamine, and hydroxyethyl starch. The evaluation encompassed three distinct medical conditions: congestive heart failure, hypotension, and hypertension. The system demonstrated effective responsiveness across all test scenarios in the simulation, successfully regulating hemodynamic signals to realign with the desired target values. 

An automated closed-loop control system proposed by Wassar T. et al has demonstrated that it is able to keep MAP near target and its performance is superior to that of manual control of infusion [43]. Similar controller systems were able to acceptably titrate different vasopressors (norepinephrine vs. phenylephrine) in the simulated patient model after adjusting for the anticipated differences between the two agents [44]. 

Maintaining hemodynamic stability in brain death (BD) donors is essential for preserving the quality of donated organs for transplantation. Manual stabilization is challenging due to the absence of vasomotor function in BD donors. Closed-loop stabilization offers a way to enhance the availability of suitable donor organs, indicating feasibility in less demanding patient populations. A dynamic model representing the pharmacology of nitroglycerine was identified using an experiment involving an anesthetized pig, employing a gradient-based output error approach for controller design. This model was then utilized to create a robust PID (proportional integral derivative) controller aimed at preventing hypertension. The controller's effectiveness was assessed in a subsequent experiment involving another brain-dead pig. Simultaneously, hypotension was prevented through closed-loop control of noradrenaline infusion, utilizing a controller previously published in the literature. The study demonstrated the feasibility of the investigated modeling and control synthesis approach in maintaining normotension in a porcine BD model [45]. A summary of the clinical trials on closed-loop systems to control blood pressure is given in Table 1.

**Table 1:** Summary of Randomized Controlled Trials on Closed-Loop System for Blood Pressure Control.

Author/Year	Study Title	Sample population	Intervention and control groups	Primary Outcome	Findings	Conclusion
Ngan Kee W. D. et al. (2013) [24]	Randomized comparison of closed-loop feedback computer-controlled with manual-controlled infusion of phenylephrine for maintaining arterial pressure during spinal anaesthesia for caesarean delivery.	Term parturients, (n=222) for elective Caesarean delivery under spinal anaesthesia.	Intervention group- systolic Arterial pressure (AP) maintained near baseline with phenylephrine infusion by computer-controlled infusion utilizing a proportional algorithm (n=108) vs. control group (n=103)- manual-controlled infusion utilizing an on-off algorithm.	The number of interventions required to manage AP.	The number of interventions was greater in the manual-control group (P,0.001), but there were no between-group differences in other outcomes.	Closed-loop feedback computer-controlled phenylephrine infusion provided better AP control.
B. L. Sng et al. (2014) [25]	Closed-loop double-vasopressor automated system vs manual bolus vasopressor to treat hypotension during spinal anaesthesia for caesarean section: a randomized controlled trial	Women with singleton full-term pregnancies, presented for elective caesarean section under spinal anaesthesia.	Intervention group (n=106)- automated vasopressors (phenylephrine and Ephedrine) Administration per algorithm vs. Control group (n=107)- administration by anesthesiologist.	Performance errors (percentage, median, and median absolute)- differences between measured and baseline BP- calculated by formulas.	The automated vasopressor group had lower median absolute performance error vs. control and reduced incidence of nausea. Other outcomes were similar in both groups	Automated systems afforded better control of maternal blood pressure and reduced nausea with no increase in reactive hypertension vs. manual boluses.
Ngan Kee Warwick D. et al. (2017) [26]	Closed-Loop Feedback Computer-Controlled Phenylephrine for Maintenance of Blood Pressure During Spinal Anesthesia for Cesarean Delivery: A Randomized Trial Comparing Automated Boluses Versus Infusion	Women with, singleton pregnancy, age ≥18 years, for elective Cesarean delivery under spinal anesthesia	Computer-controlled Bolus group (n=102) vs. Computer-controlled Infusion group (n=102).	Performance errors (percentage, median, and median absolute)	Median absolute performance error was less in the bolus group vs. the infusion group. In the bolus group, phenylephrine consumption was smaller; this was associated with smaller values for median performance error. There were no differences in cardiac output, nausea or vomiting, or neonatal outcomes between groups.	Blood pressure control was more precise when computer-controlled phenylephrine was delivered using intermittent boluses rather than continuous infusion.
Warwick D Ngan Kee et al. (2017) [27]	Performance of a closed-loop feedback computer-controlled infusion system for maintaining blood pressure during spinal anesthesia for cesarean section: a randomized controlled comparison of norepinephrine versus phenylephrine.	Patients (n=104) were scheduled for elective cesarean section under spinal anesthesia.	Computer-controlled Norepinephrine infusion group vs. Computer-controlled Phenylephrine infusion group.	Median absolute performance error.	The median absolute performance error was smaller in the norepinephrine group versus the phenylephrine group. In addition, the median performance error was smaller and the wobble was smaller in the norepinephrine group versus the phenylephrine group.	The precision of the control of blood pressure was greater with norepinephrine compared with phenylephrine at the drug concentrations used.
Joostenet al. (2021) [30]	Automated closed-loop versus manually controlled norepinephrine infusion in patients undergoing intermediate- to high-risk abdominal surgery: a randomized controlled trial	Adult (>18 years) patients undergoing intermediate- to high-risk abdominal surgery, who required advanced cardiac output monitoring and careful arterial pressure control.	Intervention group (n=15) Closed-loop vasopressor system (CLV) vs. control group (n=15).	Percentage of time that a patient was hypotensive, defined as mean arterial pressure (MAP) <90% of their baseline value, during surgery.	The percentage of time patients were hypotensive during surgery was 10 times less in the CLV group vs. the control group. The CLV group also spent much less time with MAP <65 mm Hg).	Computer-assisted adjustment of norepinephrine infusion significantly decreases the incidence of hypotension compared with manual control.
Joosten et al. (2021) [33]	Computer-assisted Individualized Hemodynamic Management Reduces Intraoperative Hypotension in Intermediate- and High-risk Surgery: A Randomized Controlled Trial	Adult patients scheduled for elective intermediate to high-risk surgery were expected to be managed according to an individualized hemodynamic protocol using a radial catheter coupled to an advanced hemodynamic monitoring device.	Computer-assisted -Intervention group (n=19) vs. manually adjusted GDT group-control group (n=19)	Intraoperative hypotension is defined as the percentage of intraoperative case time patients spent with a MAP < 90% of the patient’s baseline value, measured during the preoperative screening.	Intraoperative hypotension was 1.2% in the computer-assisted group vs. 21.5% in the manually adjusted goal-directed therapy group. Mean SVI and cardiac index were both significantly higher in the computer-assisted group (p<0.001).	In patients having intermediate to high-risk surgery, computer-assisted individualized hemodynamic management significantly reduces intraoperative hypotension.
Desebbe et al. (2022) [34]	Control of Postoperative Hypotension Using a Closed-Loop System for Norepinephrine Infusion in Patients After Cardiac Surgery: A Randomized Trial	Patients>18 years old undergoing elective cardiac surgery, in ICU	Intervention group (n=20) vs. control group (n=17) (postoperative CLV vs. manual management group).	The primary outcome was the percentage of the study period (the first 2 postoperative hours in the ICU) during which patients were hypotensive, defined as a MAP <65 mm Hg.	The percentage of time with hypotension was significantly lower in the CLV group than that in the control group.	Closed-loop control of norepinephrine infusion significantly decreases postoperative hypotension compared to manual control in patients admitted to the ICU after cardiac surgery.
Merouani et al. (2008) [40]	Norepinephrine weaning in septic shock patients by closed-loop control based on fuzzy logic	Septic patients in ICU with at least one organ dysfunction and on norepinephrine infusion (either at the clinician's discretion (control group) or under closed-loop control based on fuzzy logic (fuzzy group).	Fuzzy (intervention) group (n=19) vs. control group (n=20).	Time to cessation of norepinephrine.	The median (interquartile range) duration of shock was significantly shorter in the fuzzy group than in the control group (28.5 [20.5 to 42] hours versus 57.5 [43.7 to 117.5] hours; P < 0.0001).	The study has shown a reduction in norepinephrine weaning duration in septic patients enrolled in the fuzzy group.

Challenges 

Despite promising results, closed-loop vasopressor administration faces several challenges. The study of cardiac function has not received as much attention when it comes to closed-loop pharmacologic interventions. This can be attributed to the unique nature of the physiological effects caused by cardiac medications, such as altering heart rate or boosting heart muscle contraction strength. Many of these effects are complex and don't follow a linear pattern, and they often reach a maximum limit even when the medication is administered at low rates. There have been only a few studies conducted with inotropes, and it's clear that more clinical research is required in this area. While blood pressure is crucial for end-organ oxygen delivery, it is important to consider that an adequate cardiac output (CO) is also an essential component. Overly constricting blood vessels can raise systemic vascular resistance and reduce end-organ blood flow. To prevent this "dry vasoconstriction," optimizing CO with fluids should be pursued in parallel to vasopressor therapy. Furthermore, a comprehensive arterial pressure management system would possess the capability to adjust all these factors using medications or similar methods. Considering the complex nature of these systems with multiple inputs and outputs, along with safety and regulatory considerations, it may take some time before such a system becomes widespread. Moreover, integration of various monitoring devices and compatibility with existing ICU infrastructure can be complex and costly. Algorithms need continuous refinement to accommodate patient heterogeneity and adapt to dynamic physiological changes. Furthermore, ethical considerations surrounding the delegation of clinical decisions to automated systems and potential algorithm errors require careful attention. A significant challenge in terms of control and application lies in the complexity and transparency of the algorithm. A straightforward physiological logic would make it easier for clinicians to comprehend how the system functions and its limitations. This enhanced understanding improves safety, ultimately aiding the process of regulatory approval [46].

Closed-loop hemodynamic systems should serve as supportive tools for clinicians, never as substitutes. The human touch and qualities that we provide to patients and their families should not be replaced by machines. While clinicians can acquire the expertise to rely on artificial intelligence models, there is uncertainty about the extent to which patients will trust algorithms and how they prefer to receive algorithm-generated results. Consequently, qualitative research is essential to explore the ethical, cultural, and societal impacts of integrating AI into clinical practices.

Even though none of the experimental closed-loop systems are currently accessible to clinicians, various systems have been extensively researched and exhibit considerable potential. In order to drive forward closed-loop innovations, active collaboration with regulatory bodies, control experts, and software engineers will be crucial in the future.

Future prospects

The field of closed-loop vasopressor administration holds great potential for further advancements. Improved algorithmic models, leveraging artificial intelligence and machine learning, could enhance the accuracy of dose calculations and responsiveness to patient needs. Additionally, the integration of closed-loop systems with electronic health records and interoperability among different medical devices could streamline data exchange and enhance clinical decision-making. There is a growing interest in merging basic automated systems with the increasingly popular field of artificial intelligence and machine learning. Allowing adaptive artificial intelligence systems to control the co-administration of fluids and vasopressors would create safer, more tailored systems for unique patient scenarios, although it will demand substantial engineering and clinical collaboration. In the end, it's crucial to understand that the primary aim of these closed-loop systems is to improve the management of each patient and their clinical outcomes.

The future of closed-loop systems likely involves interconnected systems that can interpret, integrate, and optimize the key determinants that influence cardiac output and organ perfusion. Another significant development to enhance the automation of closed-loop systems will involve shifting from a responsive system to a predictive one. Closed-loop systems should incorporate safety mechanisms, including cut-off points, in case of input loss (e.g., monitoring malfunction) or treatment application issues (e.g., pump failure, pump disconnection). Nevertheless, advanced closed-loop hemodynamic systems are rapidly evolving, enhancing adherence to goal-directed strategies and potentially boosting outcomes compared to manual control. These systems should be viewed as tools supporting clinicians, with authors and developers working to enhance perioperative safety and consistency for patients.

## Conclusions

Closed-loop vasopressor administration offers potential benefits in optimizing hemodynamic stability for surgical and critically ill patients. These systems use real-time patient data and advanced algorithms for precise and individualized blood pressure control. Adoption is increasing due to the promise of maintaining consistent optimal blood pressure levels during surgery and critical care. Throughout this review, we explore a range of closed-loop approaches, from algorithms based on simple physiological principles to those incorporating advanced monitoring technologies and artificial intelligence. Each approach has demonstrated the ability to effectively titrate vasopressor infusions, resulting in improved hemodynamic stability and, consequently, better patient outcomes. Potential advantages include reducing healthcare provider workload and preventing hypotensive episodes. Moreover, the potential reduction in the workload of healthcare providers and the prevention of hypotensive episodes further underscore the clinical significance of closed-loop systems. While challenges such as technological integration and algorithm refinement persist, the potential benefits in terms of improved patient outcomes and resource utilization are substantial. The authors agree that there exists a significant potential in closed-loop systems to revolutionize certain aspects of patient care positively. Nonetheless, it's essential to consistently assess and address risks to ensure patient safety. Continued research, development, and clinical validation are essential to fully harness the capabilities of closed-loop systems and shape the future of vasopressor therapy in perioperative as well as in the critical care settings. Large-scale randomized controlled trials are still needed to establish the superiority of closed-loop systems over conventional methods in terms of mortality and morbidity outcomes. 
